# Expert-Guided Generative Topographical Modeling with Visual to Parametric Interaction

**DOI:** 10.1371/journal.pone.0129122

**Published:** 2016-02-23

**Authors:** Chao Han, Leanna House, Scotland C. Leman

**Affiliations:** Department of Statistics, Virginia Tech, Blacksburg, VA, United States of America; University of Memphis, UNITED STATES

## Abstract

Introduced by Bishop et al. in 1996, Generative Topographic Mapping (GTM) is a powerful nonlinear latent variable modeling approach for visualizing high-dimensional data. It has shown useful when typical linear methods fail. However, GTM still suffers from drawbacks. Its complex parameterization of data make GTM hard to fit and sensitive to slight changes in the model. For this reason, we extend GTM to a visual analytics framework so that users may guide the parameterization and assess the data from multiple GTM perspectives. Specifically, we develop the theory and methods for Visual to Parametric Interaction (V2PI) with data using GTM visualizations. The result is a dynamic version of GTM that fosters data exploration. We refer to the new version as V2PI-GTM. In this paper, we develop V2PI-GTM in stages and demonstrate its benefits within the context of a text mining case study.

## Introduction

Data visualizations offer an efficient way to assess, explore, and learn from high-dimensional, complex datasets. Thus, there are countless dimension-reduction methods for the purpose of visualization including, Principle Component Analysis (PCA), Multidimensional Scaling (MDS), Self-Organizing Map (SOM), and Generative Topographic Mapping (GTM) [1–4]. The methods differ by the assumptions and metrics used to determine the best two or three dimensions in which to display the data. In practice, analysts assess the assumptions and select one visualization approach that seems the most reasonable for the application. However, the decision is often unclear—modern datasets may have multiple and/or complicated structures that may contradict the constraints or parameterization of any one visualization approach. Slight adjustments to visualization methods may reveal new information in the data that would not have otherwise been seen. With this in mind, a form of human-computer interaction called Visual to Parametric Interaction (V2PI) [5] was developed to allow analysts to guide the parameterization of complex visualizations. Here, we develop the theory and methods to integrate V2PI with GTM.

Introduced by Bishop, C. M. et. al [4], GTM is a nonlinear latent manifold modeling approach for high-dimensional data spatialization and visualization. It is a probabilistic alternative for both SOM and Nonlinear PCA [6]. Relative to SOM, the visual capabilities of GTM are similar, but GTM overcomes many limitations of SOM [4]; e.g., GTM can preserve topographic ordering (i.e., objects close in the data space remain close in the visualized space), may account for missing data, and enables multiple model comparisons for comparing uncertainty in visualizations. However, GTM has some of its own shortcomings. One, the procedure used for fitting GTM to data challenges most practitioners. There are many sensitive tunable parameters which can dramatically affect the model fit. Two, the parameters in GTM are global so that local structures are hard to find by GTM. For example, if a dataset includes subclusters within large clusters, GTM may uncover the large clusters, but may fail to display the subclusters. Three, GTM is an automated modeling procedure. Given optimal parameter values, analysts assess the data from only one (GTM) perspective.

To improve GTM and make it more flexible, we extend the automatic GTM algorithm to a visual analytics (VA) framework so that users may guide the GTM parameterization. Fundamental to VA is the notion that “interaction is insight” [7]. When users interact with a visualization, no matter the capacity, users have the potential to make sense of the data efficiently and assimilate new information with old. V2PI is one form of interaction (Section 1.1). V2PI allows users to inject domain-specific information into a visualization and adjust the parameters of display-generating algorithms or models by *only* interacting with the data. For example, users may filter the data, drag observations together or apart, and/or cluster a subset of observations to suggest a re-weighting of observations or variables in the underlying display-generating model or algorithm. The advantage of V2PI is that users need only understand the data and how to interpret the display to make complex parametric changes. However, the theory and methods must be in place to interpret data interactions quantitatively and update the parameters in response. For this paper, we develop V2PI for GTM so that we have a dynamic, user guided version of GTM. We refer to the new version of GTM as “V2PI-GTM.” We demonstrate the clear benefits of V2PI-GTM in both applied and simulated examples.

The remainder of this paper is organized as follows. We provide detailed background on V2PI and GTM in Section 1. In Section 2, we merge the two to develop our method V2PI-GTM. In Section 3, we apply V2PI-GTM to explore information in a text dataset from the National Institute of Health (NIH). We then complete our paper with a conclusion in Section 4.

## 1 Background

### 1.1 Visual to Parametric Interaction

Data visualizations have matured from static graphs and dashboard widgets to interactive graphs where users may adjust images directly (e.g., with Photoshop) or the parameters that created the images. For example, interactive PCA (iPCA) [8] allows users to change data images by adjusting the data eigen-space via dials of eigenvalues; and XGvis [9], an interactive MDS visualization software, allows users to change the weight of dimension dissimilarities via sliders. Direct adjustments of parameters has its advantages, but such adjustments can lose utility when analyzing high-dimensional data or using complicated models. Most users can interpret visualizations, but not the parameters in the models that created the visualizations. This is particularly true for GTM.

Thus, we consider a form of interaction called Visual to Parametric Interaction (V2PI) [5]. In V2PI, users guide display-generating parameters by interacting only with the data. The idea is that, if users change a display to reflect what they know (or conjecture) about the data, the display-generating parameters need to be adjusted as well. Thus, the machinery of V2PI quantifies the user interaction within the display and updates the parameters based on the quantifications. Formally, the steps involved for V2PI are as follows: 1) characterize the data by a model or algorithm to reduce the dimension for visualization, 2) Visualize the data, 3) users assess the visualization and communicate their expertise or “provide feedback” about the display by interacting with it (e.g., by adjusting the positions of observations), 4) interpret the feedback quantitatively and tune the model parameters to reflect the feedback, and 5) reconfigure the visualizations based on the updated parameters.

V2PI is most successful when users only participate in step 3; i.e., users only interpret and interact with the data in a visualization. Thus, software is needed to implement steps 1, 2, 4, and 5. To build the software, however, we need the theory and methods to model or summarize the data in a reduced dimensional form, parameterize feedback, and update the display-generating parameters. Since GTM is an insightful approach to visualize data, we develop V2PI theory and methods for GTM. In the next section, we describe GTM in detail.

### 1.2 The GTM Model

**Fig 1:**
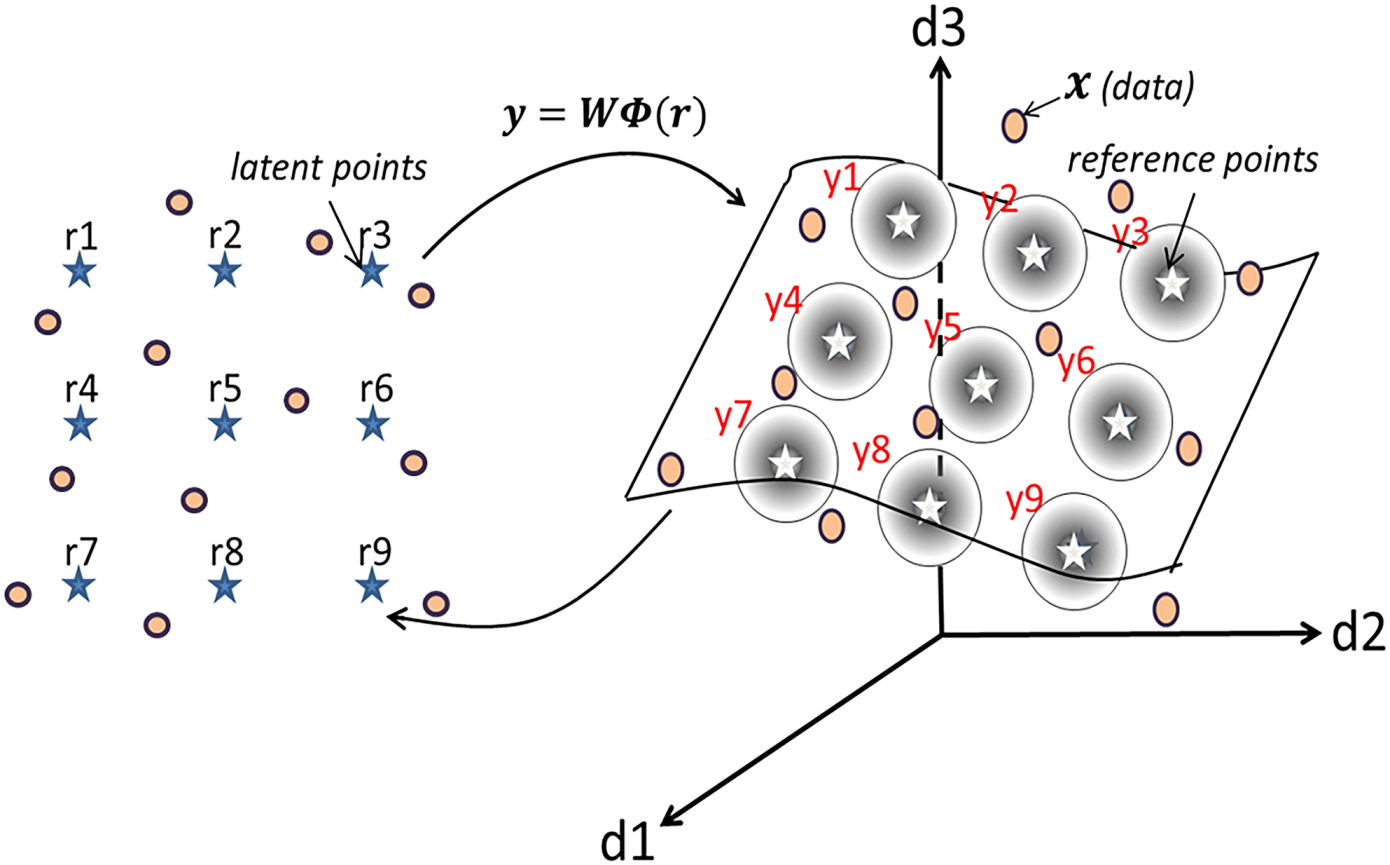
A visual description of GTM. This exemplifies how the latent space constructed by ***r*** (denoted by ⋆ on the left) and the manifold constructed by ***y*** (denoted by ⋆ on the right) in a three-dimensional data space relate. Raw data points ***x*** are denoted by •.

GTM is a non-linear latent variable model, where the latent variables define a manifold that is bent and/or twisted to embed in the high-dimensional data space. The latent space is arbitrary in dimension (so long as it is smaller than the data space), but it is usually two-dimensional for visualization purposes and summarized by a two-dimensional lattice ***r*** = [*r*_1_, …, *r*_*J*_] (*q* × *J* matrix, *q* = 2 usually), as shown on the left hand side of Fig 1. Latent points ***r*** are nonlinearly mapped to reference points ***y*** = [*y*_1_, …, *y*_*J*_] (*p* × *J* matrix, *p* is the data dimension) which sit on the manifold in the high-dimensional data space. Given the manifold, we model each observation in the data ***x*** = [*x*_1_, …, *x*_*N*_] (*p* × *N* matrix) by a Multivariate Gaussian distribution, with mean *y*_*j*_ (*j* ∈ {1, …, *J*}) and precision matrix **I**_*p*_*β* so that

$$p\left(x_{i}|y_{j},\beta \right)={\left(\frac{\beta }{2\pi }\right)}^{p/2}\text{exp}\left(-\frac{\beta }{2}{\left\|x_{i}-y_{j}\right\|}^{2}\right),$$ (1)

where ‖ ‖ denotes the Euclidean norm. The right hand side of Fig 1 shows a three-dimensional manifold example, where ***y*** (denoted by ⋆) are the centers of the radial symmetric Gaussian distributions (denoted by balls).

The nonlinear mapping in GTM takes the format of a linear regression model, in which *y*_*j*_ is estimated by a linear combination of a set of fixed *K* basis functions, such that,

$$y_{j}=W\Phi \left(r_{j}\right),$$ (2)

where W is a *p* × *K* transformation matrix. The basis function *Φ*_*k*_(*r*_*j*_) (for *k* ∈ 1, …, *K* and *j* ∈ 1, …, *J*) represents a radially symmetric Gaussian kernel,

$$\Phi_{k}\left(r_{j}\right)=\exp\left(-\frac{\|r_{j}-\mu_{k}{\|}^{2}}{2\sigma^{2}}\right),$$ (3)

where *σ*^2^ designates the spread of the radial functions and ***μ*** = [*μ*_1_, …, *μ*_*K*_] (*q* × *K* matrix, *K* is the number of basis functions) are the Gaussian centers which are typically selected to cover the latent space ***r*** uniformly. In this paper, we call the vector functions **Φ**() = [Φ_1_(), …, Φ_*K*_()] “attractors” since they define the degree to which similar points in the high-dimensional space attract toward one another in the low-dimensional visualization.

The GTM parameters are estimated via maximum likelihood estimation as described in [4]. By doing so, the objective is to find the optimal manifold described by ***y*** that a) approximates the structure of the observed data points ***x*** well (as determined by the data likelihood) and b) when unraveled and flattened by inverting Eq (2), creates a reasonable visualization of the high-dimensional data. In the visualization, one reasonable low-dimensional coordinate is selected for each observation *x*_*i*_ based on the posterior multinomial distribution of *r* given the data *x*_*i*_, where *r* can take any value in the lattice {*r*_1_, …, *r*_*J*_} with probabilities {*R*_*i*1_, …, *R*_*iJ*_}; i.e., *r*|*x*_*i*_ ∼ multinomial(*R*_*i*1_, …, *R*_*iJ*_). Each posterior probability or “posterior responsibility” (as termed in Bishop et. al [4]) equals

$$R_{ij}=p\left(r_{j}|x_{i}\right)=p\left(x_{i}|r_{j}\right)/\sum_{j^{\prime }=1}^{J}p\left(x_{i}|r_{j^{\prime }}\right).$$ (4)

Given estimates for *R*_*i*1_, …, *R*_*iJ*_ for *i* ∈ {1, …, *N*}, GTM may plot any summary of *r*, including the posterior expectation of *r*, the posterior mode of *r*, or a posterior quantile of *r*. Typically, the posterior expectation is plotted.

**Fig 2:**
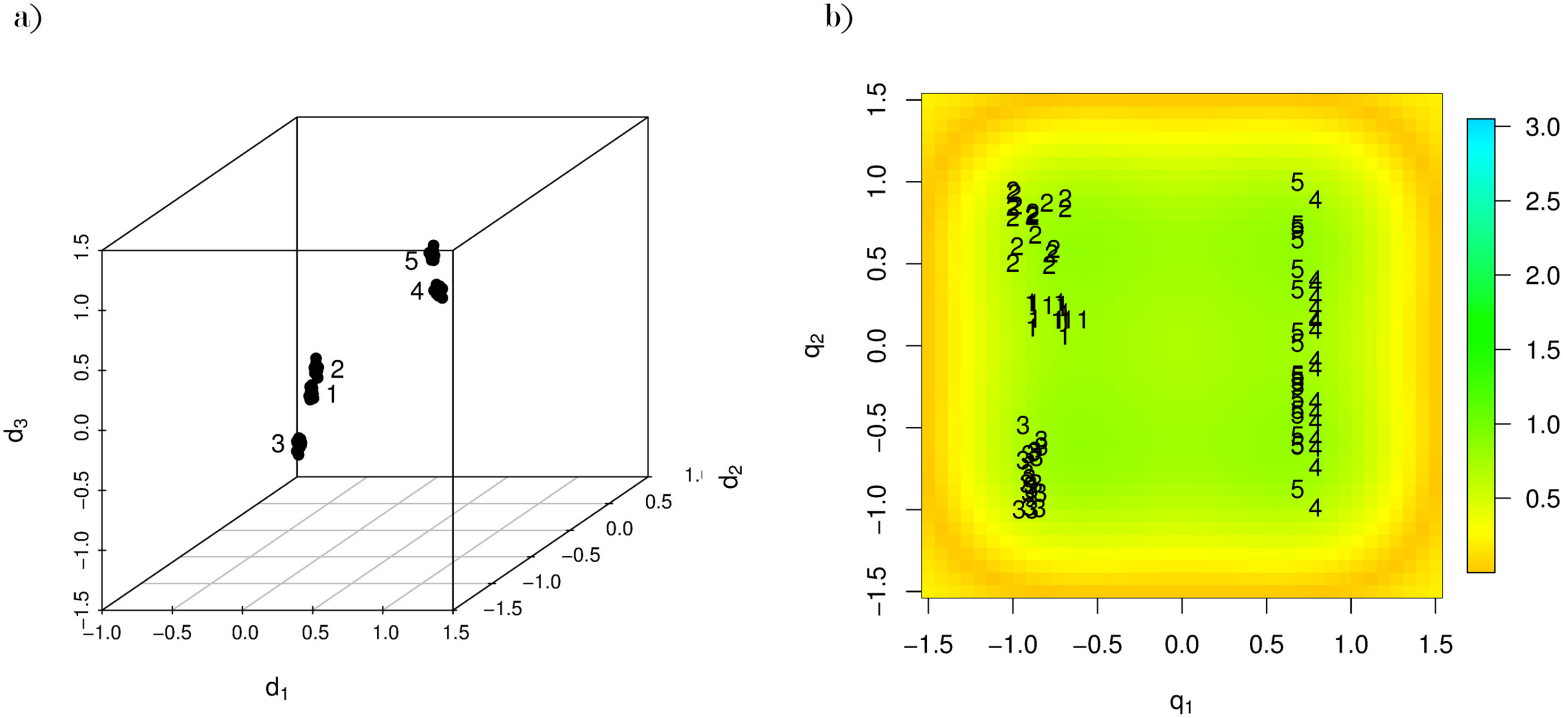
A simulated three-dimensional dataset from five Multivariate Normal distributions and its GTM visualization. Fig a) shows that there are two groups of clusters in three dimensions. The first group includes clusters 1, 2, and 3. The second group includes clusters 4 and 5. Fig b) provides a two-dimensional visualization of the data using GTM.

GTM may preserve topological ordering in that observations close or distant in the high-dimensional space appear close or distant, respectively, in the visualization. To see this, we simulated a three-dimensional dataset from five different Multivariate Normal distributions and apply GTM. Fig 2a plots the raw data and shows five clusters; there are two groups of clusters that are located in opposite corners of the three-dimensional space. When we apply GTM, with *K* = 16 and *J* = 400, and plot the posterior expectation of *r* for each observation, we obtain Fig 2b. Notice that even though GTM is not a formal clustering algorithm, GTM roughly maintains the cluster structure and clearly separates the two groups of clusters. This is because GTM preserved topographical ordering. Observations within a cluster are similar to one another high-dimensionally, and thus appear close to one another in a GTM visualization.

However, high-dimensional distance is measured along the manifold, so topological ordering is not guaranteed and the interpretation of “close” (i.e., distance between observations) in a GTM visualization requires explanation. Unlike linear methods, such as PCA and MDS, the meaning of one unit in distance is not necessarily uniform across a GTM view. For example, suppose two pairs of low-dimensional points, *r*_*a*_, *r*_*a*′_ and *r*_*b*_, *r*_*b*′_ are equi-distant from one another (dist(*r*_*a*_, *r*_*a*′_) ≈ dist(*r*_*b*_, *r*_*b*′_)), but appear in different regions of a GTM visualization. How we infer the degree to which the observations *x*_*a*_ and *x*_*a*′_ are similar (or different) to one another, relative to the relationship between *x*_*b*_ and *x*_*b*′_, depends upon the high-dimensional manifold. Crudely, if the high-dimensional manifold is flat, dist(*x*_*a*_,*x*_*a*′_)≈dist(*x*_*b*_,*x*_*b*′_). Whereas, if (*x*_*a*_, *x*_*a*′_) are separated by hills, valleys, and/or twists in the manifold and (*x*_*b*_, *x*_*b*′_) are not, dist(*x*_*a*_,*x*_*a*′_)>dist(*x*_*b*_,*x*_*b*′_); observations (*x*_*b*_, *x*_*b*′_) are more similar to one another than (*x*_*a*_, *x*_*a*′_). To help with the interpretation of distance in GTM, Bishop et. al [4] suggest color-coding a “magnification factor” that reflects the slope of the high-dimensional manifold at locations in the a GTM display [10]. In Fig 2a, we include the magnification factor. Green and yellow represent flat and erratic regions in the manifold, respectively.

Also, the current parameterization of GTM could not separate all of the clusters well, and GTM, in its current form, is not flexible. It would be hard for typical users of GTM visualizations to make worthwhile parametric changes. Thus, in the next section, we extend GTM to respond to user guidance via the visualization.

## 2 Methods: V2PI-GTM

Although GTM has tremendous advantages, it has two main pitfalls. One, there is a limit to which GTM may fold and twist a data manifold [11]. The parameters impact the model fit globally so that it is difficult for GTM to uncover meaningful local structures. Two, GTM has many tunable parameters that are hard to interpret and may have big influences on the manifold (hence visualization). Combining one and two, typical analysts do not know how to adjust GTM parameters to adjust the manifold and create new summaries of the data.

To overcome the pitfalls and foster making sense of data with human-data interactions, we develop V2PI-GTM. With V2PI-GTM, users can guide the complicated GTM parametrization and assess data from varying perspectives by just interacting with a visualization. In this section, we develop V2PI-GTM in three stages. At each stage, we make an improvement to GTM with user interactions, but identify an issue that we address and overcome in the next stage. The third and final stage describes our complete version of V2PI-GTM. We use the simulated data from Section 1.2 to exemplify each stage.

### 2.1 Stage 1, Basic V2PI-GTM Set-up

**Fig 3:**
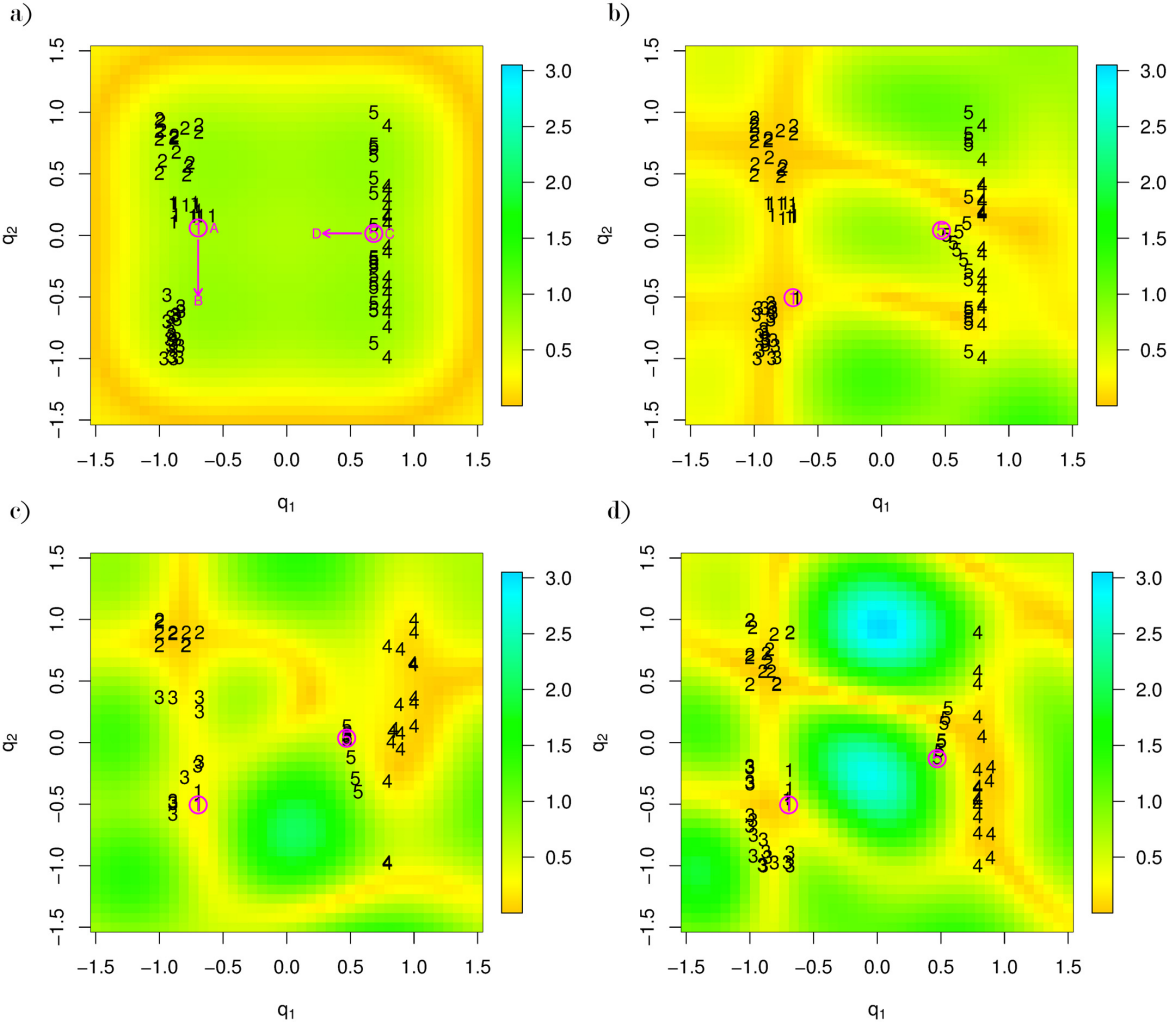
The progression of V2PI-GTM. Fig a is a GTM display (in latent dimensions *q*_1_, *q*_2_) of the simulated dataset when *K* = 16, *J* = 400. The data points are labeled according to their cluster numbers. The arrows show how a user may interact. A user may move one point from location A to location B and another point from location C to location D. Figs b, c, and d show respectively how the observations respond (or do not respond) to the move when stages 1, 2, and 3 of V2PI-GTM are in place.

As we described in Section 1.2, GTM is an nonlinear modeling approach that spatializes data in a visualization so that distance between observations has meaning. Thus, one natural form of interaction is to drag one or more observations so that the spatialization changes; i.e., the low-dimensional distances between the selected point and the remaining points change. For example, a user could drag an observation from location A to B, as shown in Fig 3a. Now, we develop V2PI-GTM to quantify the meaning of an adjusted spatialization and update the parameters of GTM to create a new display of the data.

When users select observations to move, we expand the GTM model by two parameters and give users control over them. To explain, suppose a user selects and adjusts the coordinates of one low-dimensional point that represents x* in the high-dimensional dataset *x* = [*x*_1_, …, *x*_*N*_]. To interpret the adjustment, we first add to the GTM model a low-dimensional coordinate r* that maps directly to the selected observation x* (by setting the posterior responsibility defined in Eq (4) that r* generates x* via y* to 1). Then, coupled with r*, we add an attractor Φ*() to GTM. This means that we expand sets ***r*** and **Φ** (as defined in Section 1.2) so that *r* = [*r*_1_, …, *r*_*J*_, r*] and **Φ**() = [Φ_1_(), …, Φ_*K*_(), Φ*()], where $\Phi^{*}\left(\right)=\exp\left(-\frac{\|r_{j}-\mu^{*}{\|}^{2}}{2\sigma^{2}}\right)$ and μ* = r*. By making the additions to GTM, users specify parameters r* and μ* by moving the low-dimensional coordinates of the selected observation x*.

Conditional on the specifications for r* and μ*, the GTM machinery estimates the remaining parameters and plots the data accordingly. For example, the matrix W expands by a row and is estimated by maximizing the likelihood. For this reason, observations similar to x*, as defined by the new manifold, should appear close to r* as it is moved in the visualization and far, otherwise. For example, with V2PI-GTM in place, we select a point from our simulated data at location A of Fig 3a and move the point to location B. As we see in Fig 3b, one observation follows. Similarly, we move a point at location C to D. Those most similar follow again.

Ideally, all of the observations within the clusters of the moved points would shift in Fig 3b. They do not in part because of drastic changes in the manifold, as reflected by the differences in the magnification factors of Fig 3a and 3b. When *K* is large (*K* = 16, in this case), there is enough flexibility in the model to add hills and valleys to the manifold to maintain the original layout of the data. The attraction provided by the new Φ* may not be strong enough to overcome these new hills and valleys; Φ* cannot compete with all of the other attractors. This suggests that Stage 1 V2PI-GTM is sensitive to *K*, the number of attractors.

In the next section we improve V2PI-GTM. We reduce the impact that *K* has on GTM visualizations.

### 2.2 Stage 2, Scaling

In the second stage, we keep the basic model set-up for V2PI-GTM in that we a) allow users to adjust the location of an observation and b) we expand the GTM model to include new components r* and μ*. Now, however, we change the way by which GTM updates in response to user specifications for r* and μ*.

We develop a method that is similar in spirit to local regression [12] for high-dimensional data. We modify the generative Gaussian distribution in Eq (1) (for which y* is the Gaussian center), so that it is a function of ‖x* − *x*_*i*_‖. That is, we scale *x*_*i*_ − y* in Eq (1) by the square-root of a scaling function *V*() which depends upon Δ_*i*_ = ‖x* − *x*_*i*_‖/*h*,

$$p\left(x_{i}|y^{*},\beta \right)={\left(\frac{\beta }{2\pi }\right)}^{p/2}\text{exp}\left(-\frac{\beta }{2}V\left(\Delta_{i}\right){\left\|x_{i}-y^{*}\right\|}^{2}\right),$$

where *h* is user-defined. From our experience, effective *V*(Δ) functions include Δ, Δ^2^, Δ^3^, $\exp(-\frac{1}{\Delta })$ and $\exp(-\frac{1}{\Delta^{2}})$.

This local regression scaling works in the following way. When a data point *x*′ is near x* in the data space, *V*(Δ′) will be small and, due to the increase of the posterior responsibility that r* generates *x*′ Eq (4), the coordinates for *x*′ will pull toward x* in the two-dimensional view. Similarly, points that are far from x* in the data space will push further away. The degree to which points push and pull depends on *h*. We found that good specifications for *h* relate to how many observations users expect will follow or are similar to the selected observation x*. We recommend that *h* equals a distance such that the number of nearest neighbors within *h* from x* equals the expected number. For example, if a user wants to make sure that x* attracts at least 3 matches, *h* equals the distance between x* and the third nearest data point.

To show the effectiveness of including *V*(Δ′), we apply GTM to the simulated data again. We also move the same point shown from location A to B (Fig 3a). With *V*(Δ) = Δ^3^ and *h* set to 20, we see how the data respond to the adjusted observation in Fig 3c. In particular, notice that, unlike Fig 3b, all the points in cluster 1 move. Similarly, when we move a point from location C to D. Again, all of the points in its cluster move.

Unfortunately, there are two drawbacks for this stage of V2PI-GTM. First, the transition from Fig 3a to 3c is not smooth. There can be abrupt changes in the visualization, no matter what scaling function *V*() we use. Second, except for the points that are similar to the adjusted point r*, all of the observations drift away from their original places. With this side effect, data explorations could be become complicated. Users could loose track of the relationship between the new cluster locations and their original locations, so that a sequential data exploration becomes hard to develop.

The drawbacks are due to global changes in the manifold *y*. The way by which GTM is parameterized currently results in users making global manifold changes when they only want local. In the next section, we improve V2PI-GTM again.

### 2.3 Stage 3, Mixtures of Manifolds

To minimize abrupt changes in the stage 2 V2PI-GTM, we extend GTM to include mixtures of manifolds. Let $y_{j}^{\left(c\right)}$ and $y_{j}^{\left(u\right)}$ represent the current and user-adjusted manifolds, respectively. We define the V2PI-GTM estimate for the manifold, $y_{j}^{\left(c+1\right)}$, by

$$y_{j}^{\left(c+1\right)}=\delta_{j}y_{j}^{\left(c\right)}+\left(1-\delta_{j}\right)y_{j}^{\left(u\right)},$$

where *δ*_*j*_ = ‖*r*_*j*_ − r*‖/*b* and *b* = max{‖*r*_1_ − r*‖, …, ‖*r*_*m*_ − r*‖} so that *δ*_*j*_ ∈ [0, 1]. This definition for $y_{j}^{\left(c+1\right)}$ controls the visualization so that only the regions of interest respond to user interactions. Points in the areas that are distant from dragged observations do not change their positions.

We apply one final application of V2PI-GTM to the simulated data. Again, *K* = 16, *V*(Δ) = Δ^3^ (*h* = 20), and we move the same set of points from location A to B and from location C to D. Now, however, we have mixtures of manifolds in the GTM model. The visualization updates smoothly as r* moves. In the final view (Fig 3d), the circled points are able to attract the data points which belong to their respective clusters. The other clusters stay at their original positions, except for several observations from cluster 3 that are repelled (as they should be).

### 2.4 Discussion

This final stage of V2PI-GTM describes our complete approach to allow users to explore data from multiple perspectives with GTM by dragging observations. By dragging, we give analysts access to the following parameters in GTM: a latent point r* in ***r*** and basis function Φ*() in **Φ**(). Although, we increase the number of parameters in GTM, we shelter users from the mathematics of GTM and slightly reduce the challenge of selecting good specifications for parameters *J* and *K*. Users must make a “judged trade-off” between computational resources and visual resolution when specifying *J* and *K* [10]. However, by our methods, *J* and *K* adjust (i.e., increase by one) each time users interact with the data. Subsequently, new visualizations are created that rely on different weights of the high-dimensional variables and the visualizations evolve smoothly as user insights develop.

That said, when expert judgement is included in analyses, there is often an active choice in the degree to which it may impact analytical results. Here, users may adjust observations one by one in V2PI-GTM and, with each adjustment, the role of expert judgement in the visualization expands. In fact, experts could adjust every observation in a dataset to specify an arbitrary spatialization. Although users may still learn from GTM by “tagging” (defined below) when they adjust all observations, users lose the opportunity to discover structure in the data that they did not already know. Consumers of V2PI-GTM should be aware of this, and consider taking measures to maintain the high utility of GTM. Specifications for ideal measures is an active research area. For now, we recommend only moving a minor fraction of observations that are relevant to research questions and hypotheses. Additionally, we remind readers that V2PI-GTM is not an inferential methodology, but one that supports data exploration.

At face value V2PI-GTM might seem similar to a visual analytic method called Dust and Magnets (DnM) that was developed by Yi et. al [13]. In DnM, users drag or shake nodes that represent variables in the dataset and watch as relevant observations (denoted by particles of iron dusts) follow the nodes. However, V2PI-GTM differs from DnM in two fundamental ways. First, the nodes that users adjust in V2PI-GTM represent individual observations, not variables. Thus, users need only to understand the relationship between two or more observations to inject their expertise or conjectures about the data into the visualization. Users are not expected to know the relative importance of entire dimensions in the dataset. Second, when users drag observations they are effectively comparing all (not two) of the variables in the dataset simultaneously, relative to the observation moved. In this simulated example, the three dimensions work in combination to define or break clusters. If we wanted, we could use features of GTM to “tag” the latent space and assess differences in the combinations between updated visualizations.

To tag the latent space means to label selected points in the latent space by values of the high-dimensional variables. We provide examples of tagging in Section 3.1. Tagging is possible because V2PI-GTM maintains a relationship between the GTM parameters and data visualizations by the V2PI process. That is, updated visualizations are a direct result of updated model parameters that define a manifold. We use tagging to interpret the manifold in terms of the original variables. This enhances V2PI-GTM as a tool to explore datasets and assess them different, interpretable perspectives. In the next section, we explore text data by tagging the latent space and applying V2PI-GTM.

## 3 Application: Text Mining

Exploring a collection of documents can be a time consuming, complex task. Often analysts use keyword searches or document matching [14] to identify patterns in the dataset. Searching for keywords (and the documents that contain them) is simple and fast, but lacks rigor. For example, keyword searches may identify documents with similar keywords, but used in different contexts; miss documents that contain combinations of the keywords; or prioritize words inappropriately for the purposes of the data exploration. Document matching, on the other hand, groups documents based on many keywords, phrases, and/or query topics. It is an improvement over keyword searches, but can be hard to implement. GTM-V2PI provides a natural, interactive, and visual way to document match.

In this section, we illustrate the application of V2PI-GTM within the context of text mining. We have a collection of 54 abstracts with 2365 entities (words) from proposals funded by the National Institute for Health (NIH) [15]. Based on the abstracts, suppose NIH Program Managers want to assess the allocation of funds to varying research areas. However, the definitions of ‘research areas’ are ambiguous; e.g., goals of proposals can overlap regardless of the fields with which principal investigators may associate. For this reason, we apply V2PI-GTM to explore the data and learn about the NIH priorities, as reflected by similarities and dissimilarities among the proposal abstracts.

Before we apply V2PI-GTM, however, we pre-process the data. First, we apply standard text mining procedures to remove uninformative entities, such as, stop words or redundant words (e.g., run and running become run). Second, we rank the entities using a new algorithm that we call the Imp-Index (ImpI) and select those that are top ranked. ImpI is described in Appendix A and based on the Gini coefficient [16] that measures relative importance of entities in datasets, typically text datasets. With our pre-processing, we transform the original count data to continuous ImpI measures and reduce the dimensionality from 54 × 2365 to 54 × 1000.

**Fig 4:**
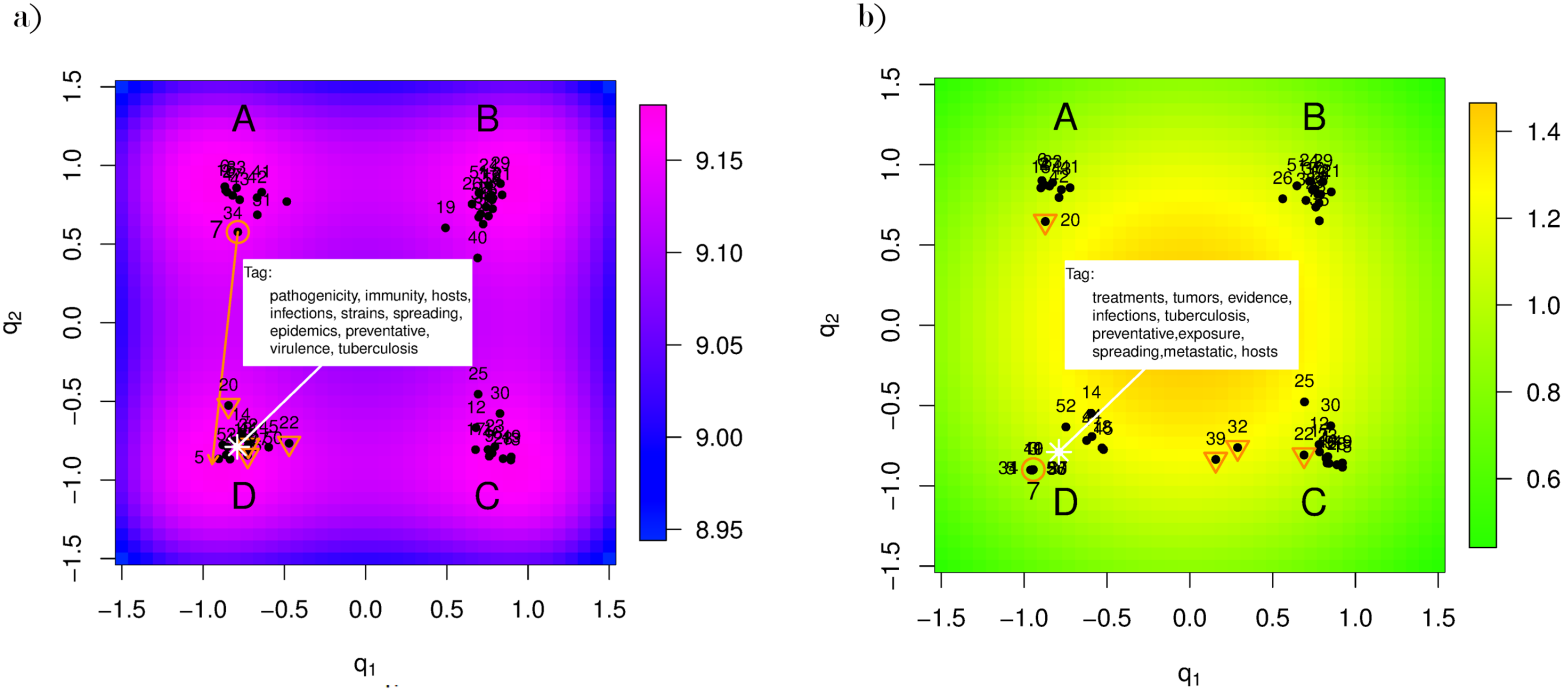
V2PI-GTM with NIH data. We provide a GTM display of the NIH abstracts (labeled by their identification numbers) before and after user interaction in Figs a and b, respectively. The interaction is portrayed by the pink arrow in Fig a; Abstract 7 was moved to a location near cluster D. In addition, to labeling and learning about four clusters in the data (marked by A, B, C, and D), we also tagged the latent GTM space. After the interaction, we see that the clusters grouped differently and the meaning of the latent space changed. Also, the manifold changed dramatically.

Based on the ImpI metric, spatial visualization methods, such as GTM, could be used to explore text data. Thus, we apply GTM for *K* = 16 and *J* = 400 to obtain an initial display of the proposals, shown in Fig 4a. Notice a manifold with hills and valleys separates the data into four clusters. We label the clusters A, B, C, and D. Suppose that a program manager has particular interest in Abstract 7 (highlighted in pink in Fig 4a) from cluster A. Abstract 7 is about developing new brain tumor therapies and tumor stem cell quiescence. The program manager would like to assess how many and in what ways other granted proposals were similar to Abstract 7. But first, it helps to describe the latent space.

### 3.1 Describing the Latent Space

In this scenario, the program manager would like to explore the data with a direct intention: to learn about similarities and differences in the data relative to abstract 7. Whereas, in Section 2, we simply assess how the data respond to moved observations—any move and any observation. The intent was undirected. For complicated datasets, repeated undirected interactions could inform users about the number of structures in the data, the types of structures in the data, the sensitivity of the data to GTM, etc. For users with directed intent, e.g., to compare specific documents, it is helpful to describe observed data structures in visualizations and the latent space in terms of the raw variables (in the high-dimensional space).

For example, to understand how the clusters differ, we determine the words that both overlap the least within each cluster and have the highest ImpI’s. We first apply a common clustering algorithm, known as k-means [17], to the low-dimensional data coordinates to determine cluster memberships. For each cluster, we sum the ImpI vectors across the documents and rank the entities based on the ImpI sum. Entities ranked highest are those that 1) have importance in the corpus and 2) have occurred most frequently in the document cluster. Given a top-ranking termlist from each cluster, we delete those shared by all four clusters so that we have unique key words that describe each cluster. We list these words in Table 1. Cluster A represents proposals that include brain related cancer studies and their clinical applications. Cluster B is about studies related to human neural systems; e.g., stem cells, neuro-degenerative diseases, human pluripotent and neural circuits. Cluster C represents proposals that address genomic and transcriptomic research problems. Cluster D represents proposals about infectious diseases, such as tuberculosis, and immunity.

**Table 1:** This table lists the Top 10 keywords that either differentiate clusters A, B, C, and D or are shared among all of the clusters in Fig 4.

Cluster A	tumors, brains, stem, treatments, patients, generations, drugs, ordering, controlling, therapeutics
Cluster B	stem, neuronal, brains, proteins, deliveries, regulations, neural, patients, differentiation, expression, treatments
Cluster C	stem, genetically, regulations, drugs, structurally, proteins, genomics, epigenetics, RNAs, complexities
Cluster D	Infections, treatments, tuberculosis, expression, patients, drugs, strains, resistance, vaccination, immunity
Shared	cells, functionalization, diseases, developments, genes, cancerous, studying, researchers, proposing, mechanisms, specification

Knowing how the clusters differ is helpful and correlates with the meaning of the latent space around the clusters. That is, varying regions in visualizations reflect weighted combinations of the high-dimensional variables. These combinations are derived directly from V2PI-GTM that we summarize by tagging. To demonstrate, we select spot, *r*^+^, in the visualization and use Eq (2) to estimate its corresponding location on the manifold, *y*^+^. In this application, estimate *y*^+^ is a 1000 × 1 vector of ImpIs. All or a subset of the 1000 entities could be reported as a visualization tag; we select ten. For example, in Fig 4a, we pick a spot *r*^+^ (represented by a pink circle) that locates roughly at the center of cluster D. According to its corresponding location on the manifold, the top ten keywords include: pathogenicity, immunity, hosts, infections, strains, spreading, epidemics, preventative, virulence, tuberculosis. As expected, several of these words overlap with the words describing cluster D.

Based on the meanings of cluster A, cluster D, and *r*^+^ in Fig 4a, a program manager may learn what he/she needs from the current visualization or re-organize the dataset by applying V2PI-GTM. In the next section, Abstract 7 is relocated.

### 3.2 Cluster reorganization of NIH dataset

From the previous section, we know that Abstract 7, shares the following keywords with cluster A: tumors, brains, cancerous, therapeutics, and chemotherapy. However, Abstract 7 also shares some keywords with cluster D; e.g., treatments, strategies, patients, drugs, resistance, clinically. Suppose that the program manager is particularly interested in the latter set of key words. Thus, the manager drags Abstract 7 to the lower left corner of the display (shown by the pink arrow in Fig 4a) near the location of cluster D and observes how the remaining documents react in Fig 4b.

As expected, many documents in cluster D gravitate toward Abstract 7 or stay close to their original locations in Fig 4b. However, some repel. Abstracts 20, 22, 32 and 39 that were originally in cluster D, relocate to new regions of the visualization. Table 2 describes each abstract. Abstracts 20 and 22 repelled from Abstract 7 and shifted respectively to cluster A and C because the redefined-manifold down-weighted their shared entities with the original cluster D and up-weighted their unshared entity tumor. Similarly, Abstracts 32 and 39 separated slightly from cluster D and gravitated toward cluster C because they have a few keywords in common with each cluster, but not enough to place them in either corner.

**Table 2:** Descriptions of Abstracts 20, 22, 32 and 39 in Fig 4.

20	discusses diagnosis of HIV infection in patients who live with limited access to therapeutic treatments
22	discusses expression characteristics of a drug-resistant gene
32	discusses varying yeast strains
39	discusses Lymphocyte Homing

To further understand the re-clustering of the abstracts, we assess changes in the manifold. Overall, the magnification factor is lower in Fig 4b than in Fig 4a which suggests that the new manifold is flatter than the original. Clusters in Fig 4a are located in hilly regions on the manifold, whereas, clusters in Fig 4b are in flat, stable regions. This suggests that there is a combination of variables such that the observations surrounding Abstract 7 are more similar to one another than originally suggested. We can assess the combination by tagging, as shown in Fig 4b. Notice the tag differs from that in Fig 4a.

Given Fig 4b, the program manager could have reason to return to and re-asses the original clustering of the data or continue exploring the data from varying perspectives with V2PI-GTM. He/she could select other abstracts to compare and contrast or consider an undirected exploration of the data. As in any data exploration, it is the program manager’s choice.

## 4 Conclusions

Among current visualization algorithms, GTM has had success in visualizing unstructured data [11, 18, 19]. It is robust to outliers and offers more flexibility than standard linear projection methods. However, GTM is complicated by its extensive parameterization, which often prohibits exploration by direct parametric interaction.

In this paper, we modified the original GTM to a) take advantage of its strong visualization capability, and b) overcome its drawbacks. Albeit, there are versions of GTM, such as [20], and other feature extraction methods that analyts could use to discover structure in data, but we propose a method within an interaction framework. By interacting with datasets and exploring them visually from different perspectives, analysts gain insight efficiently and dynamically. Thus, we develop V2PI-GTM whereby analysts may organize observations directly in a display and watch how the GTM machinery relocates the remaining observations in response. In a way, V2PI replaces the role of quantitative experts and protects users from the mathematics of data analyses. The communication between users and models is through the visual metaphor. We show the utility of V2PI-GTM in a text mining application.

Future work in V2PI-GTM would be to enable more interaction, in addition to dragging. For example, it would be interesting to explore the parameterization of interactions, such as, filtering, linking, highlighting, and zooming. We could also parameterize the dragging of multiple data points at one time (rather from sequentially). The more ways we can provide users to interact with GTM, the more expert knowledge we can inject into data explorations. Also, since we maintain a probabilistic framework in GTM, subsequent analyses that require formal inferential statements (e.g., inferences with assessments of uncertainty) are a natural progression following a data exploration with the probabilistic version of our framework.

## Appendix

### A Importance Index (ImpI)

ImpI is similar to the commonly used term frequency—inverse document frequency (tf-idf) [21] in the sense that it considers both the frequency and uniqueness of words that are shared across documents. Consider an term *e*_*i*_ that occurs *f*_*ij*_ times in document *d*_*j*_. The ImpI for *e*_*i*_ is given by

$$ImpI_{i}=\frac{\sum_{j=1}^{N}\sum_{k=1}^{N}\left|f_{ij}-f_{ik}\right|}{2N^{2}\mu_{i}},$$

where N is the total document number and $\mu_{i}=\frac{\sum_{j=1}^{N}f_{ij}}{N}$ is the average frequency for term *e*_*i*_. ImpI ranges between 0 and 1. Entities that occur equally frequently in all the documents have ImpI = 0 and entities that occur in only one document has ImpI = 1. ImpI can be used to rank and hence filter terms. We selected the 1000 entities with the highest ImpI’s and describe each proposal by a 1000 × 1 term frequency vector with frequency *f*_*ij*_ weighted by ImpI. The weighted frequency is defined as $f^{*}=ImpI_{i}\times \frac{f_{ij}}{\|d_{j}\|}\times \frac{\|e_{i}\|}{F}$, where $\|d_{j}\|=\sum_{i=1}^{K}f_{ij}$, $\|e_{i}\|=\sum_{j=1}^{N}f_{ij}$ and $F=\sum_{i=1}^{K}\sum_{j=1}^{N}f_{ij}$. For document *d*_*j*_, the weighted vector is given by $f_{j}^{*}=\left(f_{1j}^{*},\ldots ,f_{Kj}^{*}\right)$; the vector element equals zero when the proposal does not include the entity.
